# Teleost Metamorphosis: The Role of Thyroid Hormone

**DOI:** 10.3389/fendo.2019.00383

**Published:** 2019-06-14

**Authors:** Marco António Campinho

**Affiliations:** Centre for Marine Sciences (CCMAR), Faro, Portugal

**Keywords:** thyroid hormones, metamorphosis, teleost, morphogenesis, asymmetry

## Abstract

In most teleosts, metamorphosis encompasses a dramatic post-natal developmental process where the free-swimming larvae undergo a series of morphological, cellular and physiological changes that enable the larvae to become a fully formed, albeit sexually immature, juvenile fish. In all teleosts studied to date thyroid hormones (TH) drive metamorphosis, being the necessary and sufficient factors behind this developmental transition. During metamorphosis, negative regulation of thyrotropin by thyroxine (T4) is relaxed allowing higher whole-body levels of T4 that enable specific responses at the tissue/cellular level. Higher local thyroid cellular signaling leads to cell-specific responses that bring about localized developmental events. TH orchestrate in a spatial-temporal manner all local developmental changes so that in the end a fully functional organism arises. In bilateral teleost species, the most evident metamorphic morphological change underlies a transition to a more streamlined body. In the pleuronectiform lineage (flatfishes), these metamorphic morphological changes are more dramatic. The most evident is the migration of one eye to the opposite side of the head and the symmetric pelagic larva development into an asymmetric benthic juvenile. This transition encompasses a dramatic loss of the embryonic derived dorsal-ventral and left-right axis. The embryonic dorsal-ventral axis becomes the left-right axis, whereas the embryonic left-right axis becomes, irrespectively, the dorsal-ventral axis of the juvenile animal. This event is an unparalleled morphological change in vertebrate development and a remarkable display of the capacity of TH-signaling in shaping adaptation and evolution in teleosts. Notwithstanding all this knowledge, there are still fundamental questions in teleost metamorphosis left unanswered: how the central regulation of metamorphosis is achieved and the neuroendocrine network involved is unclear; the detailed cellular and molecular events that give rise to the developmental processes occurring during teleost metamorphosis are still mostly unknown. Also in flatfish, comparatively little is still known about the developmental processes behind asymmetric development. This review summarizes the current knowledge on teleost metamorphosis and explores the gaps that still need to be challenged.

## Introduction

Teleosts (ray-finned fish) constitute the most diversified vertebrate group (1). They comprise more than 23,000 species and occupy a wide range of aquatic habitats, morphologies, behavior, and physiology. Most teleosts develop indirectly, i.e., between the end of embryonic development and sexually immature juvenile stages, they assume a transitional larval form where rudiments of all organs are already present, although not mature. Also, teleost larvae can present different ecologies and physiologies from their adult form.

It is only at the transition from larvae to juvenile that the metamorphosis occurs, and where the larva develops into a fully formed fish identical to the adult form but still sexually immature. In most teleosts, with both symmetric and asymmetric morphologies, this developmental transition is orchestrated by thyroid hormones (TH) that are the sufficient factors necessary for larvae to undergo metamorphosis. Production of TH occurs in the thyroid gland and consists of the prohormone thyroxine (T4) and the active hormone triiodothyronine (T3). Conversion, transport, and binding of T3 to its cognate receptors are tightly regulated at the cellular level since TH needs to be in a strict physiological range (2).

**Figure 1:**
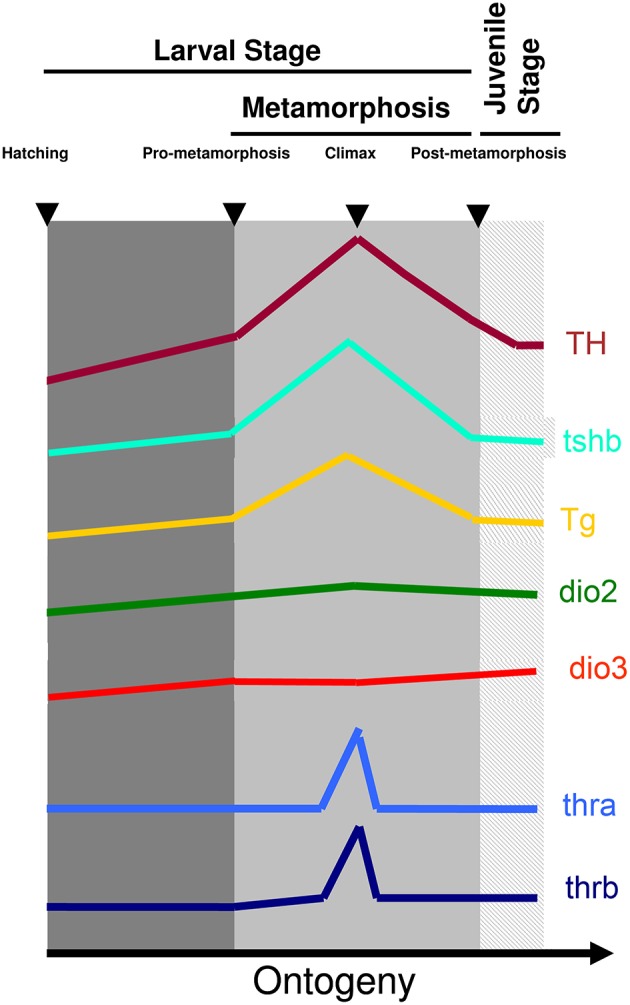
Archetypal profile of T4 and T3 and expression of *tshb, tg, dio2, dio3, thra*, and *thrb* genes during teleost metamorphosis. The general observation in teleost species so far indicates that a surge of TH is accompanied by a rise in *tshb* and *tg* expression and the increased expression of TH signaling genes *dio2* and *thyroid hormone receptors* together with a decrease of *dio3*. As soon as metamorphosis terminates TH levels decrease concomitantly with decreased levels of expression of *tshb* and *tg* and *dio2* and *thyroid hormone receptors*, whereas *dio3* levels increase to pre-metamorphic levels. Figure adapted from (15) with permission from Elsevier.

The involvement and dependence of TH for teleost metamorphosis were initially found in flatfish (3–15). Later it was also found that in most symmetric teleost species, metamorphosis occurs and parallels the developmental landmarks seen in flatfishes. The pre-metamorphic stage is characterized by lower whole-body levels of T4 and T3 and lower expression of *thyrotropin* (*tshb*), *thyroglobulin* (*tg*), *deiodinase 2* (*dio2*), and *thyroid hormone receptor beta* (*thrb*) and higher expression of *deiodinase 3* (*dio3*) (Figure 1). As soon as metamorphosis started, T4 and T3 increase together with increased expression of *thsb, tg, thrb*, and *dio2* and decreased *dio3* expression (Figure 1). The levels of T4 and T3 and expression of *tshb, tg, thrb, dio2* peak at the climax of metamorphosis, whereas *dio3* expression attains its lowest expression levels (Figure 1). As metamorphosis terminates the levels of T4 and T3 and markers of gene expression return to pre-metamorphic levels (15–24) (Figure 1). So far the observed markers and stages of metamorphosis are conserved between teleosts and anurans, clearly showing that this is a homologous developmental process regulated by TH (Figure 1).

The evidence today points to TH regulation of most organ maturation and developmental processes that occur during teleost metamorphosis. These changes enable not only a more efficient locomotion and digestion but also physiological and metabolic adaptations that allow the juvenile fish to adapt to their new habitat and lifestyle.

## Central Regulation of Metamorphosis

One of the outstanding characteristics of anuran and teleost metamorphosis, in comparison to other developmental events, is the existence of a central regulation at the organismal level together with organ/tissue/cell-specific regulation of TH signaling. This regulation enables metamorphosis to occur when appropriate environmental conditions are achieved. A better example remains unknown where the factor that regulates each developmental event is also regulated at the central organismal level so that increased serum concentration can drive specific cellular developmental events.

**Figure 2:**
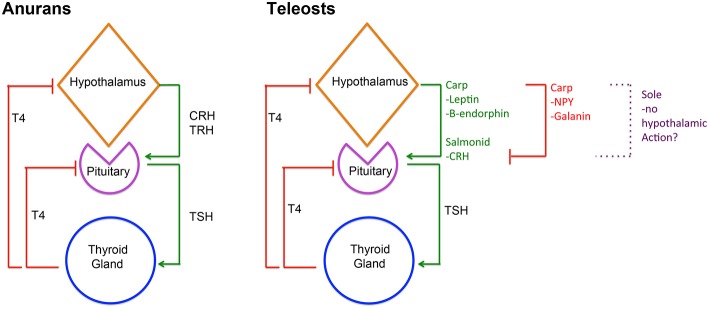
Diagram depicting HPT-axis regulation in anurans and teleosts. In anurans, hypothalamic derived CRH and/or TRH are involved in regulating pituitary TSHb expression and secretion that in turn regulates T4 production in the thyroid gland and consequently serum levels. Inversely, serum T4 then negatively regulates CRH/TRH and TSHb expression and secretion. In teleosts, HPT-axis regulation is more diverse and likely reflects species specificity. In salmonids, CRH seems to regulate pituitary TSHb expression and secretion, whereas in carp leptin and β-endorphin seem to assume that role. Notably, in carp, Galanin, and NPY seem to repress TSHb expression and secretion. Remarkably, in sole metamorphosis, neither TRH nor CRH seems to be involved in regulating pituitary TSHb raising questions about the role of the hypothalamus in HPT-axis regulation in these teleosts. Despite this, in all teleosts studied so far, the negative feedback loop between thyroid gland T4 and pituitary TSHb is present and conserved.

Given the importance of TH in the regulation of a wide range of molecular pathways, their production by the thyroid gland is tightly controlled by the hypothalamic-pituitary-thyroid (HPT) axis, which ensures homeostasis of TH serum levels. This serum TH homeostasis is achieved in different ways in vertebrates so far studied. In adult mammals, hypothalamic thyrotropin-releasing hormone (TRH) is released into the hypophyseal portal system and regulates the production of thyrotropin (TSHb) by the pituitary gland, that in turn regulates TH production by the thyroid gland (25). In adult reptiles and birds, the hypothalamic factor, corticotropin-releasing hormone (CRH), has a more prominent role in regulating thyrotropin (TSHb) secretion and T4 serum levels than TRH (26, 27). However, in teleosts, the current knowledge suggests species-specific regulation of the HPT axis (28–34). The observation that in adult cyprinids, Leptin, Galanin, β-endorphin, and neuropeptide Y (NPY) can regulate *in vitro* pituitary *tshb* expression (Figure 2), suggests that in teleosts pituitary-thyroid regulation may occur by hypothalamic factor/s other than TRH or CRH or by other, non-hypothalamic, endocrine factors (34) (Figure 2). Some studies in teleosts suggest that hypothalamic inhibition rather than stimulation by an unidentified factor might constitute the primary mechanism for HPT-axis regulation (35–37). In flatfish, even considering the known involvement of TH in metamorphosis, the knowledge on the regulation of the HPT-axis and the underlying neuroendocrine regulation remains very scarce.

During post-embryonic development, TH are the necessary and essential factors regulating metamorphosis. TH exerts their effect through a whole-body and tissue/cell-specific manner, both in anurans and flatfishes (3–5, 27, 28, 38, 39). In metamorphosing anurans, the central regulation of metamorphosis involves both the hypothalamic-pituitary-thyroid (HPT) axis and the hypothalamus-pituitary-adrenal gland axis (HPA), more commonly associated with stress (Figure 2). Environmental cues, such as decreased water levels, stimulate the HPA axis, and the release of CRH, which acts via its receptor, CRHr2, in pituitary thyrotrophs to enhance TSHb secretion, TH production and triggering metamorphosis [revised in (27); Figure 2]. A common feature of anurans and flatfishes is the simultaneous increase in *tshb* expression and TH serum levels during metamorphosis. In anuran metamorphosis, the set-point of the HPT axis is modulated by the action of CRH on pituitary thyrotrophs, so that high levels of serum TH do not repress *tshb* expression [revised in (27)]. In adult teleosts and during metamorphosis, TH are the main regulators of pituitary *tshb* expression, pointing to a central negative feedback mechanism at the level of the pituitary and the thyroid gland (28, 31, 32, 34, 40, 41) (Figure 2). During flatfish metamorphosis, hypothalamic inhibition is relieved, and the negative feedback on pituitary *tshb* by plasma TH adjusts to a higher set-point (28, 30, 41). Goitrogens block sole (*Solea senegalensis*) metamorphosis, indicating that the negative feedback loop between the thyroid and the pituitary gland is functional in larvae well before metamorphosis (28, 41, 42). Evidence on how the flatfish hypothalamus regulates the HPT-axis remains elusive. Recent work on flatfish metamorphosis, suggests that hypothalamic thyrotroph regulation may not exist at all during sole metamorphosis (41) (Figure 2). In sole larvae, blocking of metamorphosis after methimazole treatment (that blocks iodination of Tg and T4 production) did not change the temporal and spatial expression of *trh* and *crh*, suggesting that these neuroendocrine factors are not involved in sole metamorphosis. As a whole, the evidence raises fundamental questions about hypothalamic regulation of TH during metamorphic development in teleosts. The way *tshb* expression is regulated to induce metamorphosis of symmetric teleosts is even less well-known, and a great gap of knowledge exists on how the onset of metamorphosis occurs in these teleosts. It is still one of the great open questions in teleost metamorphosis.

Collectively, these evidences highlight a conserved mode of action in teleosts and an integrated response of the larval organism to the signal of TH. First, *tshb* level increases whole organism TH content that in turn gives rise to local tissue/cell responses. These are mediated locally by *dio2* and *dio3* ratios (discussed below) and increased *thrb* expression that allows the initiation of the metamorphic program and the morphogenetic changes in sensitive tissues. As in anurans, in teleost, metamorphosis *thrb* is considered to be the major *TH receptor* mediating metamorphic cell responses. However, there is little to none functional evidence in teleosts to support this view. This consideration derives from evidence in anurans [revised in (27)] and the existence, by whole-body analysis, of a peak in expression of *thrb* at the climax of metamorphosis in several teleosts (13–16, 43, 44). Since in most teleost species there are at least three different *TH receptor* genes (*thraa, thrab*, and *thrb*), it is likely that different *TH receptors* are involved in specific metamorphic events. The most likely scenario is that in some cells/tissues, metamorphic morphogenetic events are regulated by *thraa, thrab*, or *thrb* or even a combination of all or some *TH receptors* in a given cell/tissue. Nonetheless, in sole metamorphosis, the asymmetric development of the pseudomesial bone (discussed below) is correlated only with the asymmetric expression of *thrb*, strongly suggesting that, at least for this metamorphic event, *thrb* is the main effector of TH function (45). The actual scenario on the TH receptors mediating each metamorphic event is not clear and more work is necessary to elucidate this question in teleost metamorphosis.

## Morphological, Morphogenetic, and Physiological Changes During Teleost Metamorphosis

The word metamorphosis derives from the ancient Greek, where it means a change in form. In teleosts, just as in anurans, the larvae undergo such morphological changes that the overall shape of the animal gives rise to the juvenile form. In flatfish, this developmental event results in a dramatic morphological change. The symmetric flatfish larva develops into an asymmetric juvenile that is characterized by the migration of one eye to the opposite side of the head and the tilting of the body axis toward the migrating eye side. In the end, the primary body axis changes with regards to the original embryonic established dorsal-ventral and left-right axis (LRA). The lateral ocular side becomes dorsal, the blind lateral side becomes ventral, and the dorsal and ventral sides become left or right. The metamorphosed flatfish juvenile becomes a benthic animal in contrast to the pelagic larvae from which it arises. Its form adapts perfectly to life in the bottom of aquatic environments where it uses stealth to prey and escape predators. The morphogenetic changes in symmetric teleosts are more subtle and do not change the embryonic derived body axis of the animal.

Nonetheless, in both teleost groups, they render the post-metamorphic juvenile a more hydrodynamic efficient form (46–50), reducing drag and enabling faster and less energy consumption during locomotion. These hydrodynamic changes are accompanied by a change in swimming mode with a passage from a larval C-shape movement to a more S-shape movement (48, 50). As the larvae undergo metamorphosis, these changes preconize an adaptation of form to fit a new function/lifestyle. To enable these changes, all the major organ systems in the larvae develop in response to higher metamorphic TH-levels.

## Axial Muscle

Simultaneously with the change in locomotion, TH during metamorphosis reshapes the constitution of muscle fibers and the myotome. In most teleosts studied so far, a generalized increase in muscle hyperplasia occurs in the myotomes at the start of metamorphosis, that afterwards, gives way to increased muscular hypertrophy. This shift in muscle growth is more evident in the most epa- and hypaxial regions of the myotomes of metamorphic larvae, where there is a steep increase in the number of new muscle fibers developing (51–55, 55–66). During seabream (*Sparus aurata*) metamorphosis, several sarcomeric genes undergo isoform switching that are likely involved in modulation of locomotion (55, 55–57, 63, 64, 67). The increase in thyroid hormones during metamorphosis correlates with mRNA splicing events of *troponin T* (*tnnt*) genes in *S. aurata, Paralichthys olivaceus* (summer flounder), *Solea solea, Scophthalmus maximus* (turbot), and *Hyppoglossus hyppoglossus* (halibut) (59, 60, 68–70). Similar responses to T3 of other sarcomeric genes and proteins, like *myosin heavy chain* (71) and *Ca-ATPase* (72), are found in mammals. Some teleost species, during metamorphosis, develop a teleost-specific axial muscle denominated pink muscle that presents biochemical and functional characteristics intermediate of white and red muscle (54, 56, 73–76).

Notably, studies on halibut metamorphosis show that *dio2* and *3* play a critical role in these muscular developmental changes. The enzymes, *dio2* and *3*, are expressed in the same hyperplasic cells in the most epaxial and hypaxial regions of the myotome of early metamorphosing larvae, indicating that a tight regulation of cellular TH is essential for this cell proliferation. From the climax of metamorphosis onwards, the epa- and hypaxial cells co-expressing *dio2* and *dio3* become scattered throughout the myotome and resemble muscle pioneer cells (39). This evidence shows that TH is involved in axial muscle development during and after teleost metamorphosis. Nonetheless, more detailed studies are still needed to directly demonstrate the exact role of TH in teleost skeletal muscle development. Notably, the TH-dependent changes in skeletal muscle development in teleosts mirror some of the effects of TH in mammalian skeletal muscle development, where Dio2 and Dio3 activity is pivotal in the regulation of TH-action [revised in (77)].

## Blood

Together with locomotion changes and the joint development of axial muscle during metamorphosis, there are also changes in gas-exchange. In most teleosts, blood develops fairly soon during embryonic development (78, 79). However, embryonic/larval erythrocytes are not fully mature. Most of the gas-exchanges in larvae until metamorphosis occur by simple diffusion through the skin (80). It is only at metamorphosis that erythrocytes can efficiently retrieve CO_2_ from cells and deliver oxygen to the tissues. TH stimulates and promotes the switching in the expression of *alpha-globin* genes from larval to adult isoforms (5, 81–84). This switching enables a more efficient gas exchange in juvenile rainbow trout in comparison to larval animals, with greater Bohr effect in adult haemoglobins (82). During amphibian metamorphosis, there is a similar regulation of haemoglobins, and the evidence seems to suggest that this event occur so that the metamorphosed animal can adapt to the new post-metamorphic environment (85, 86).

Taken together, gas-exchange at the gill level becomes more prominent as metamorphosis progresses. This transition in gas-exchange is especially important since the skin becomes more complex, stratified and impermeable to gases (80, 87, 88) as a direct consequence of TH-driven skin development during metamorphosis.

## Skin

The skin of teleost larvae is composed only of an epidermal layer and has little or no stratification. As the larvae undergo metamorphosis, the skin develops into a stratified epithelium with a multi-layered epidermis and dermis, which is formed by an acellular layer of collagen that is invaded by fibroblasts (87–92). At the same time, the adult pigmentation pattern starts to develop (93–97). Interestingly, in flatfish pigmentation becomes asymmetric when eye migration terminates. However, melanocyte precursors cells are found symmetrically distributed in the animal, but only the ones on the ocular side differentiate into melanocytes (95, 96). It is still unclear how these cells respond asymmetrically to TH. In zebrafish, as metamorphosis comes to an end, scales start to develop from specialized fibroblasts that have invaded the collagen lamella (98).

In teleost skin, keratinocytes are direct targets of TH. Before metamorphosis, halibut larval keratinocytes only express *dio3* but as soon as the larvae enter the climax of metamorphosis, *dio2* becomes highly expressed (39). Together with this increase of *dio2* expression, keratinocytes start to lose their larval morphology, larval keratin isoforms are repressed and apparently, no epidermal keratin is expressed in adult body skin keratinocytes (87, 99, 100). Notably, as keratinocytes differentiate toward an adult phenotype, they lose a distinct basal keratin bundle that is present only in larval cells (87).

During the much better studied amphibian metamorphosis, it is known that both cellular and molecular changes in the skin are dependent on TH and gave rise to fully stratified skin after metamorphosis (101–103), thus paralleling the observed events in teleost skin during metamorphosis. The most striking difference between teleost and anurans during skin metamorphic development is the apparent lack of expression of post-metamorphic keratin isoforms in teleost skin [88, 103, 104].

Taken together, TH during metamorphosis gives rise to an orchestrated series of developmental events in the skin of teleosts that make this organ develop from a simple to a complex structure that is a more impermeable and selective barrier. Importantly, these changes give rise to a dramatic shift in the physiology of the larvae since gas exchanges switch from the skin to the gills (80).

## Skeletal Development

In mammals, TH is an important factor in bone development and homeostasis [revised in (104)]. So far, this aspect of metamorphosis has not received much attention in teleosts. Teleost skeleton develops significantly during metamorphosis. During the larval stage, most cartilaginous structures are already present, but most of the dermal derived bones are still absent. Most dermal derived bones develop as metamorphosis starts, and most of the cartilaginous derived bone starts to transit from their cartilaginous scaffold into an ossified definitive structure during metamorphosis (11, 45, 105–118).

Especially notable during metamorphosis is the development of the dermal derived structures of the axial skeleton, namely the vertebral bodies. These dermal derived structures start to ossify as soon as metamorphosis begins. Simultaneously, the neurocranium starts its ossification at this stage from the mesenchyme tissue surrounding the brain. Together with the resorption of the larvae fin-fold, the fin rays begin to develop and ossify, enabling the full development of the fins after metamorphosis (11, 105–107, 110–118).

One interesting aspect of how skeletal development during metamorphosis can be adjusted to allow better adaptations to the habitat conditions of juveniles, comes from *Oreochromis mossambicus* (*Mozambique tilapia*). In this species rearing of metamorphosing larvae at higher temperatures promotes carnivore-oriented skull development, whereas lower temperatures reared larvae developed omnivore oriented structures (116). Although the exact role of TH signaling is unknown, these observations highlight that during tilapia metamorphosis, skeletal development is adjusted to match the specific environmental needs posed to the larvae.

However, most of the studies on the role of TH on teleost skeletal development are descriptive. Some reports show that changes in TH signaling might correlate with abnormal skeletal development in *S. senegalensis* (113). Also, TH signaling is essential for asymmetric development of the flatfish specific pseudomesial bone (45). Despite these advances, still much remains to be found about the exact role and the genetics of TH signaling regulating bone development during teleost metamorphosis.

## Gastrointestinal-Tract

One of the key features of anuran metamorphosis is the development of the gastrointestinal (GI) tract. During metamorphosis, the tadpole gut shortens and modifies to allow a shift from herbivorous to carnivorous feeding [revised in (119)]. In mammals, TH is essential for the development of the gut, where *Thra* plays a crucial role in GI cell differentiation (120–122). During teleost metamorphosis, the larvae GI tract is also home to a series of significant morphological, molecular and functional changes (9, 123–131). In Atlantic halibut, Japanese flounder and summer flounder metamorphosis leads to higher compartmentalization of the GI tract. In these flatfish, the development of the stomach and an increased length, folding, and regionalization of the intestine is observed. Accompanying these morphological events is the beginning of the expression of *pepsinogen* (126, 129–131). Similar development of the GI tract is observed in the symmetrical teleost *Dicentrarchus labrax* (sea bass) (124, 128). One key feature of teleost intestine development at metamorphosis is the increase in microvilli area, globulet and secretory cells (124, 126, 128, 131). In halibut, one of the key features of metamorphic stomach development is the synchronous establishment of molecular and cellular components in such a way that stomach function starts at the climax of metamorphosis (126).

Together with the digestive tract changes, other organs involved in digestion respond to increased TH-levels during teleost metamorphosis. In the zebrafish pancreas at metamorphosis, β-cell differentiation and *insulin* expression increase and respond positively to exogenous TH treatment, whereas the opposite is observed for *glucagon* expression (132). In Senegalese sole, early in metamorphosis, the intestine and liver start to express *apolipoprotein A-I* that is involved in cholesterol metabolism (133). This evidence suggests that during metamorphosis, TH regulates many of the developmental events in most GI-tract organs to allow a novel metabolic and homeostatic program to start and take full advantage of the novel food sources in the post-metamorphic habitat.

## Flatfish Metamorphosis Asymmetric Head Development

Of all teleosts, flatfishes display the most dramatic morphological manifestation of metamorphosis. It was considered, since its discovery, that these teleosts become asymmetric at metamorphosis (134). The fossil record shows that eye migration is one of the early events taking place during flatfish metamorphic evolution (135, 136). Nonetheless, evidence shows that, besides skin pigmentation, asymmetric metamorphic development observed at the body level is located in the most anterior third of the head that encompasses the eye field (11, 39, 45, 59, 87, 115, 117, 118, 137, 138).

Evidence suggested that sub-dermal cell proliferation was the factor responsible for driving asymmetric eye migration during flatfish metamorphosis (139). However, in these experimental conditions of sub-ocular colchicine-inhibited cell proliferation, the eye was still able to migrate (>50%), albeit not as much as in normal developing larvae (139). This evidence suggested that, although cell proliferation is a factor involved in flatfish metamorphic eye migration, it is not the major mechanism that drives it. Later evidence in Senegalensis sole shows that asymmetric sub-ocular development and ossification of the pseudomesial bone (that develops only during flatfish metamorphosis and ventral to the migrating eye) is the major driving force of eye migration during metamorphosis (45). In methimazole treated sole larvae eye, migration was impaired, clearly showing that TH is the sufficient and necessary factor behind eye migration. Also, in these larvae, neither pseudomesial ossification nor sub-dermal cell proliferation was observed. This evidence shows that sub-ocular asymmetric ossification and sub-dermal proliferation are both TH-driven events (45). Additional evidence using sub-ocular injection of apyrase [dermal bone differentiation inhibitor (140)] in pre-metamorphic sole larvae prevented pseudomesial ossification and most of the eye migration (only 5–15% migration observed). These pieces of evidence show that pseudomesial ossification is the major force driving eye migration and asymmetric head development in sole metamorphosis (45). Given that pseudomesial bone asymmetric development at metamorphosis is a common event in all flatfish (11, 115, 117, 137, 138), the findings in sole (45) argue that this is the mechanism by which asymmetric TH-driven head development occurs in all flatfish. Together, pseudomesial asymmetric ossification and cell proliferation are essential for proper eye migration in flatfish (45, 139). In this scenario, asymmetric flatfish eye migration itself is a passive event where the eye, the eye socket, ocular muscles, and the ocular nerve are all pushed to the opposite side of the head by asymmetric ossification of the pseudomesial bone together with sub-ocular cell proliferation (45, 139). Moreover, this event compresses the mesenchyme tissue in-between the eyes that also differentiates into bone, giving rise to the paramesial bone (11, 39, 45, 59, 87, 115, 117, 118, 137, 138). The tissue mechanics underlying this process are still unknown.

The pseudomesial bone arises from an asymmetric TH-responsive center, characterized by the co-expression of *dio2* and *thrb*, that increase in expression as metamorphosis progresses and terminate as soon as metamorphosis is completed (45). In the TH-responsive center, TH regulates *dio2* in a non-canonical fashion. Rather than increased concentrations of TH leading to decreased *dio2* expression, as is observed in symmetric developing structures in the sole head; in the asymmetric TH-responsive center, *dio2* expression is dependent and positively regulated by TH (45). Nonetheless, it is still not known which are the genetic factors behind this asymmetric sub-ocular expression pattern of *dio2* during metamorphosis.

## Genetic Regulation of Flatfish Metamorphic Eye Migration

The genetic regulation of asymmetric head development in flatfish remains an elusive matter. It was suggested that flatfish metamorphic asymmetry is related to embryonic left-right (LR) asymmetric development by the *Nodal-Lefty-Pitx2* pathway (141). In both *P. olivaceus* and *Verasper variegatus*, asymmetric habenular expression of *pitx2* was found during metamorphosis of these flatfish (141). Also, it has been argued that asymmetric *pitx2* habelunar expression during metamorphosis led to the decreased size of the blind side habenula lobe that in turn generated a torsion force that would lead to both optic nerve and eye migration (141). However, no asymmetric nerve endings were shown to emerge after this asymmetric event. These observations raise questions about how *pitx2* asymmetric habenular expression can give rise to asymmetric pseudomesial ossification and cell proliferation that are the driving forces of flatfish eye migration (45, 139). Tentative efforts were also carried out to understand if blocking of *pitx2* signaling, via ouabain exposure during embryogenesis, could lead to random migration of the eye. The reversal of normal eye migration occurred in a small proportion of individuals (~6%), but the gross majority of these larvae metamorphosed normally (141).

Given that flatfish metamorphic eye migration is exclusively dependent on TH, it is still not known if *pitx2* expression in *P. olivaceus* and *V. variegatus* is dependent on TH. Moreover, *Nodal-Lefty-Pitx2* embryonic signaling was independent of putative *pitx2* asymmetric metamorphic development (141), further raising concerns about how this embryonic mechanism could lead to asymmetric metamorphic development. Notably, habenular asymmetry is not exclusive of flatfish and is also found in other bilateral teleosts (142), suggesting that the asymmetry in the habenula of *P. olivaceus* and *V. variegatus* might not be related to metamorphosis. Moreover, a detailed mapping of the nervous system during sole metamorphosis only found asymmetric differences in the olfactory epithelia, olfactory bulb and in the most anterior diencephalon that is encompassed by the eye field (45). Remarkably it was found that the olfactory epithelia and bulb only became asymmetric if there was eye migration but not in its absence. These pieces of evidence strongly suggest that the asymmetric metamorphic development of these brain structures was due to the force constraints brought about by pseudomesial asymmetric ossification. Consequently, eye migration is not directly caused by TH-action on these structures. Curiously, in zebrafish embryogenesis, it was found that maternal TH are involved in establishing *pitx2* expression in the developing pituitary but how or if this has any consequence in metamorphic *pitx2* expression in zebrafish or any other teleost species remains unknown (143).

Up until now, the most detailed data available on asymmetric flatfish head metamorphic development only sheds light on how TH regulates asymmetric pseudomesial ossification that leads to eye migration (45). Several efforts have been made using next-generation transcriptomic approaches to solve this question (144–146). However, no definitive answer was obtained even using these high-throughput strategies. Alves et al. (147) sequenced the head transcriptome of metamorphosing halibut and found that the most significant differentially expressed transcripts (DET) were related to TH production. The whole-body transcriptome of metamorphosing Japanese flounder reports that the top DET were for genes related to bone development (145). Transcriptomics confirm that increased TH production is a crucial event in teleost metamorphosis and that bone development is a central aspect of teleost metamorphosis as already shown by several studies (11, 45, 105–118). While this manuscript was in revision, a new study analyzing the transcriptome of dissected heads of metamorphosing sole larvae was published. In this study, it was also not possible to identify a single gene responsible for asymmetric head development during flatfish metamorphosis (148). Instead, it was reported that in the head of sole larvae undergoing metamorphosis, several different genetic cascades function in a time and stage-specific manner. The earliest changes are related to hormonal production, followed by protein and mRNA processing, cell cycle regulation, nuclear organization, and finally, DNA replication. Collectively, this evidence argues that metamorphosis in sole head occurs in a stepwise manner with earlier genetic cascades promoting the next stage of developmental changes. Also, this study shows that earlier occurring metamorphic events are at the organismal level while later events are at the cellular level, suggesting that metamorphosis progresses from changes in the organism to specific cellular responses (148). Nonetheless, neither study was able to identify a single gene or genetic mechanism behind asymmetric eye migration in flatfishes. These findings argue that asymmetric head development can be due to a combination of different flatfish specific genetic pathways that together give rise to asymmetric head development.

## TH and Salmonid Smoltification

In most teleosts, a single surge in TH is found and related to the larval to juvenile transition, known as metamorphosis (discussed above). However, in salmonids, a second peak of T4 also occurs just before spontaneous smoltification (24, 149). TH treatment increases survival of salmonids after SW transfer (150), further arguing that TH are involved in salmonid smoltification. The evidence seemed to suggest that T4 levels are directly related to the adaptability of salmon to seawater (SW) and parr fish that are not able to adapt to SW present hypothyroidism (150). Also, TH are involved in the acquisition of the specific morphological changes that come with smolting like silvering and behavior responses like downstream migration (151).

Little is known about the tissue-specific developmental events driven by TH during the parr to smolt transition in salmonids. An early study reported that brain and hepatic T4 levels increase before plasma levels, whereas muscle T4 content decreased as soon as plasma T4 levels increased (152). Nonetheless, the biological consequences of different organ T4 content and timing are still unknown. A more recent study provided evidence showing THs are involved in both the light response and gill seawater adaptation observed in salmons undergoing smoltification (153). In this study, Lorgen et al. (153) show that different *dio2* paralogs are involved in different tissue responsivity to TH, but the exact consequences of this differential TH cell signaling remain elusive.

Nonetheless, this evidence suggests that TH might have a role in integrating environmental cues that allow for the specific adaptions needed for seawater transition and that these changes are brought about by complex peripheral regulation of TH metabolism. Notably, T4 levels increase in wild smolts, whereas in hatchery smolts T4 levels remain stable (154), suggesting that the role of TH in salmonid smoltification is complex and likely linked to environmental cues. Albeit this evidence, and in contrast to metamorphosis that is considered a larval to juvenile transition, TH are not the necessary and sufficient factors regulating smolting. In both anuran and teleost metamorphosis, T4 and T3 increase to give rise to this developmental transition. However, instead in salmon smoltification, T4 but not T3 levels increase (24, 149, 152, 154). Taken together the evidence seems to suggest that TH participate, together with other endocrine factors [revised in (155)], in the transition from parr to smolt but they are not the essential factor triggering smoltification. As a whole, salmonid smoltification is likely to be a developmental transition specific of this class of teleost that is mediated by several endocrine factors including TH.

## Future Perspectives

The evidence so far obtained on teleost metamorphosis highlight the role of TH as an integrative factor able to give rise to a series of synchronized developmental changes across the entire organism. These prepare the organism for the physiological and ecological challenges of juvenile animals. Despite recent advances, teleost metamorphosis is still an understudied developmental event. Important questions at the core of this developmental process are still not answered. These include: (1) how central regulation of the onset of metamorphosis is achieved and; in the case of flatfish metamorphosis, (2) which are the TH-dependent genetic and cellular mechanisms behind asymmetric head development. Other aspects like the role of TH metabolites other than T4 and T3 during metamorphosis are mostly unstudied.

Also, a recent study implicated genetic divergence in *tshb* paralogs locus in mediating and modulating TH signaling and physiological adaptation in three spine-sticklebacks marine and freshwater ecotypes (156). Although no exact molecular mechanism or developmental/metamorphose-related implications of divergent TH signaling were found between the two ecotypes, these evidences also highlight the potential capacity of TH for promoting speciation in teleosts (156).

The advent and fast pace of development of new technologies will allow for better discrimination of the genetic events during teleost metamorphosis and TH signaling in general. Also, more powerful imaging technologies for large specimens open the door for discoveries and better insight into morphological development observed during teleost metamorphosis.

## Author Contributions

The author confirms being the sole contributor of this work and has approved it for publication.

### Conflict of Interest Statement

The author declares that the research was conducted in the absence of any commercial or financial relationships that could be construed as a potential conflict of interest.
